# CircRNAs and its relationship with gastric cancer

**DOI:** 10.7150/jca.32927

**Published:** 2019-10-15

**Authors:** Xinxin Fang, Jing Wen, Mingjun Sun, Yuan Yuan, Qian Xu

**Affiliations:** 1 Tumor Etiology and Screening Department of Cancer Institute and General Surgery, the First Hospital of China Medical University, and Key Laboratory of Cancer Etiology and Prevention (China Medical University), Liaoning Provincial Education Department, Shenyang, China.; 2 Department of Gastroenterology, First Affiliated Hospital, China Medical University, Shenyang City, Liaoning Province, China.; 3 Key Laboratory of Gastrointestinal Cancer Etiology and Screening, Liaoning Province, Shenyang 110001, China

**Keywords:** circRNA, gastric cancer, cell biological behaviors, diagnosis, prognosis

## Abstract

Circular RNAs (circRNAs), as a type of tissue specific RNA with more stable structure than linear RNAs, was poorly understood on its correlation with gastric cancer (GC). In this review, we outline the synthesis and characteristics of circRNAs and generalize their categories and functions. Through comprehensive analysis of the reported results, we find that circRNAs not only participate in the regulation of gastric cancer (GC) cell biological behaviors, such as proliferation, invasion, migration and epithelial mesenchymal transition (EMT), but also are related to the clinicopathological features of GC such as tumor differentiation, TNM stage and metastasis, etc. According to the present screening and verification results, circRNAs are suggested to be used as biomarkers for the early diagnosis and prognosis prediction of GC, and those circRNAs involved in the genesis and development of GC have the potential as novel targets for the individualized treatment of GC.

## Introduction

Circular RNAs (circRNAs) were first discovered by scientists when using electron microscope to observe RNA virus in the 1970s^1^. Nevertheless, due to the technique limitations, circRNAs were originally considered as by-products of mRNA error splicing, occupying minimal amount in cells^2^. With the development and progress of scientific technologies, especially the appearance of high-throughput sequencing and bio-informatics, mounting circRNA families have been found in prokaryotes, animals, plants, and humans^3^-^6^. As non-coding RNA (ncRNA) with neither 3'-5'end polarity nor polynucleotide tails, it was revealed in many researches that circRNAs play essential roles in tumorgenesis and development^7^.

**Figure 1:**
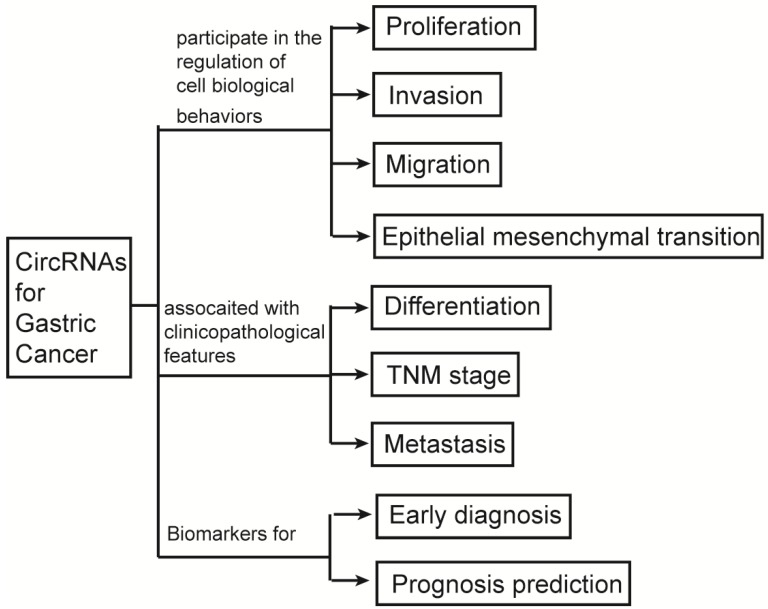
The graphic summary of this review.

Until now, a few of review articles had been previously published to elucidate the potential relationship between circRNAs and cancer. But most of them were tried to study the associated of circRNAs and the overall cancer. Compared to those of previous studies, we focused on the relationship between circRNAs and gastric cancer which is the fifth common cancer and the third major cause of cancer death worldwide 8. We aim to explore the relationships between circRNAs and GC by briefly describing the biological characteristics, process of synthesis and scavenging, classification and function of circRNAs; comprehensively summarizing the correlations of circRNAs with biological behaviors of GC cells and clinicopathological features of GC patients; and systematically reviewing the applications of circRNAs as biomarkers in prediction, diagnosis, treatment and prognosis of GC (Figure 1).

## Biological characteristics of circRNAs

On account of the unique circular structure, circRNAs vary from linear RNAs in several characteristics, which are the preconditions of being biomarkers for disease. The characteristics of circRNAs are as follow: Ⅰ. Strong stability: CircRNA is a covalently closed loop structure without 3'/5' end polarity and polynucleotide tail. Therefore, circRNA cannot be affected by ribonuclease R and RNA nucleic acid exonuclease^7^. Ⅱ. Abundant content: Owing to its strong stability and relative long half-life period, circRNA is 10 times richer than corresponding linear RNAs in cells and is also abundant in extracellular fluid^3^, ^5^. Thus, the expression levels of circRNAs in tissues at present can be detected by real-time fluorescent quantitative PCR experiment. Besides, owing to the specific and stable characteristics of circRNAs, their expression levels can also be detected in plasma, saliva and urine. Therefore, circRNAs can be regarded as promising biomarkers for diagnosis and prognosis assessment of diseases^9^-^12^. Ⅲ. Specific expression: Tissue- or developmental stage- specific expressions of circRNAs can frequently be observed in the same organism, and the specificity also exist among different species^3^, ^13^. Hence, circRNA as normal dynamic balanced product of gene and a member of regulatory pathway in cells, participates in the development of diseases and may act as specific target for the disease treatment.

## Synthesis and removal of circRNAs

The homeostasis of circRNAs in cells are attributed to their specific biosynthesis and scavenging mechanisms. Firstly, for the coding genes of circRNAs, most of their gene sequences are located in exon regions of host genes, and others are located in introns or intergenic regions^3^, ^5^, ^14^. Some exons can transcribe multiple circRNAs 3, 14. Secondly, regarding the biosynthesis patterns of circRNAs, back-splicing pattern is the most widely recognized one. The majority of circRNAs are synthesized at the post-transcriptional level through pre-mRNA back-splicing 15, 16. During the back-splicing process, the 5' and the 3' end splicing site form a covalent closed ring by 3'-5'phosphodiester bond^7^, ^14^. CircRNAs are synthesized by back-splicing at a very slow pace under natural condition, however, the biosynthetic speed can be affected by multiple factors including the competitions of pre-mRNA splicing, the distances between RNA binding protein (RBP) -induced splicing sites, the extension rates of RNA rapid polymerase Ⅱ, the controls of cis-acting elements *in vivo* and the self positive feedback, etc 15, 17-19. Besides, the known mechanism for circRNAs scavenging is that the intracellular circRNA can be excreted out of cells through the extracellular vesicles (EVs). Then, part of the EVs rupture and release the circRNAs into extracellular fluid; another part are absorbed by other cells along with encapsulated circRNAs^20^.

## Categories and function of circRNAs

Based on the coding genes, tens of thousands of circRNAs are divided into different subtypes, which are as follows. Ⅰ. exon circRNAs (ecircRNAs), generated from exons^3^. Ⅱ. intron circRNAs (ciRNAs), generated from introns^3^, ^21^. Ⅲ. exon and intron circRNAs (EIciRNAs), generated from both exons and introns^22^. Ⅳ. fusion circRNA (f-circRNAs), generated from cancer-associated chromosomal translocations^23^. EcircRNAs are enriched in cytoplasm, while ciRNAs and EIciRNAs are distributed in cytoplasm with less amount 3, 21, 24. With in-depth researches, the biological functions of circRNAs gradually emerge and researches revealed that subtypes of circRNAs were not only distinct in cellular localizations, but also in biological functions. Firstly, circRNAs can function as natural sponge of miRNAs. In cytoplasma, ecircRNAs as the sponge of miRNAs, restrain miRNAs functions, thus regulating the expressions of target mRNAs^4^, ^25^. In nucleus, ciRNAs may act as the sponge of protein, influencing cell function^17^, ^26^. Secondly, ciRNAs and EIciRNAs can activate or positively regulate the transcriptions of parental genes in the nucleus^21^, ^24^. Thirdly, as mentioned above, some cells can transport circRNAs out of cells via EVs and then were acquired by other cells through intaking these EVs, which means circRNAs may contribute to the cell communications^20^. Fourthly, latest research elucidated that intracellular circRNAs can be used as templates for protein translations. Fifthly, in cells, when the circRNAs sequence contains the m6A sequence, translation can be performed and the changes of m6A expression level affect the efficiency of this process^27^, ^28^.

## Interactive network of circRNAs with other non-coding RNA and proteins

CircRNAs can form regulatory pathways with other biomolecules in cells with miRNAs, siRNAs, lncRNAs and proteins through various different ways. Ⅰ, circRNAs could form a regulatory pathway with the small size non-coding RNA, miRNAs^4^. Hansen, TB and his team demonstrated that the target sites of miR-7 exist on ciRS-7 and the ciRS-7 can more effectively inhibit miR-7, comparing with traditional anti-miRNA methods^4^. Deserved to be mentioned, this is the first circRNA-miRNA regulatory pathway be discovered. Subsequently, scientists also revealed this ciRS-7-miR-7 co-expression pathway when they researched other miR-7 related physiological and pathological processes in the human diseases including insulin secretion, tumor, neurodegenerative diseases, intervertebral disc degeneration, systemic lupus erythematosus, etc^29^-^33^. Coexpression of ciRS-7-miR-7 was also found in the brains of different animals and plants^34^-^36^. Therefore, this circRNA-miRNA regulatory pathway not only extensively exist in nature, but also is a diversified regulatory pathway that regulates many physiological and pathological processes^34^-^36^. A miRNA may be controlled by multiple circRNAs, and a circRNA can also suppress the various miRNAs at the same time^4^, ^37^-^39^. CircRNAs can not only inhibit the function of miRNAs through the ceRNAs (competing endogenous RNAs) network mechanism to form regulatory pathways, but also affect cell function by stabilizing the miRNAs activities^40^. Ⅱ. circRNAs could form a regulatory pathway with other non-coding RNAs including small interfering RNA (siRNA) and long non-coding RNA (lncRNA). SiRNAs can infiltrate into 'RNA induced silencing complex' (RISC), leading to RNA interfere (RNAi), and then specifically inhibit the functions of circRNAs by binding with circRNAs^41^, ^42^. LncRNA or pseudogenes can inhibit the functions of miRNAs, and have synergistic effects with circRNAs 37, 43, 44. Transcription factors facilitate circRNA expressions by binding to promoters^45^. Ⅲ, circRNAs could form a regulatory pathway with proteins. The circRNAs can be the templates for mRNA translation, which impact on protein expression level directly, forming the circRNAs and proteins regulatory pathways^27^, ^28^. The regulatory pathways of circRNAs and proteins are found to be carried out in three ways. The first one is to regulate the translations of mRNAs indirectly by regulating the circRNA-miRNA pathways, thus affecting the synthesis of proteins^46^, ^47^ . CircRNA inhibits miRNA function and removes the inhibition of miRNA on mRNA, thus increasing protein content 48, 49. For example, in cells, circRNA_001569 regulates NR4A2 mRNA by acting with miR-145^50^. The second one is acting as RNA binding proteins (RBP) sponge, competitive binding with RBP, consequently influencing mRNA translation^51^. Both co-expressions of circRNA and RBP in the nucleus (such as circRNA MBL and MBL and ci-ankrd52 and POlII) and complexes formed by circRNAs and proteins in the cytoplasm are evidences of this molecular network^17^, ^21^, ^52^. Ⅳ. The above-mentioned three pattern could also function with each other which could compose of a network. A regulatory pattern that “circRNA-miRNA-mRNA” or “circRNA-miRNA-lncRNA” had been studied which were a ceRNA network^4^, ^53^. In summarized, circRNAs could form regulatory pathway with miRNAs, siRNAs, lncRNAs and proteins and then composed an interactive network.

## CircRNAs involving in the regulations the biological behaviors of gastric cancer cells

Recent researches demonstrated that the expression level of circRNAs is closely related to the biological behavior of GC cells^54^, ^55^. A study regarding GC conducted by Li,J, et al showed that circ_104916 had tumor inhibition effect, correlated with the proliferation, invasion, migration and epithelial mesenchymal transition (EMT) of GC cells^56^. In comparison with normal gastric tissues and GES-1 cell lines, the expression levels of circ_104916 in GC tissues and five GC cell lines were down-regulated^56^. And further *in vitro* experiments elucidated that circ_104916 could inhibit the proliferation, invasion, migration and the EMT process of GC cells^56^. Similarly, Zhang,J et al. found that the expression of circLARP4 in GC tissues was down-regulated compared with normal gastric tissues. circLARP4 could relieve the mRNA LATS1 inhibition by inhibiting the miR-424 function, hence affecting the proliferation and invasion of GC cells^57^. In this study, researchers first discovered that miR-424 had LATS1 binding site, facilitating the proliferation and invasion of GC cells by inhibiting the expressions of LATS1^57^. Through prediction of bioinformatics, data analysis of circRNA expression profiles and the verification of luciferase reporter assay and RNA immunoprecipitation (RIP), they came to conclusion that the circLARP4 can serve as a sponge for miR-424^57^. Further experiments were designed to explore the effects of circLARP4 on the biological behaviors of GC cells and found that overexpression of circLARP4 inhibited the proliferation and invasion of GC cells, while down-regulation of circLARP4 promoted these processes^57^. Although these studies focused on different circRNAs, they all demonstrated that the GC tissue differently expressed circRNAs were intimately related to the biological behaviors of GC cells and might play vital roles in the pathogenesis and development of GC^58^, ^59^. Thus far, still numerous circRNAs are unconfirmed, and more mechanisms concerning the impact of circRNAs on biological behaviors of GC cells remain to be explored. Association between circRNAs and gastric cancer cells biological behavior in previous studies is shown in Table 1.

## Correlation between circRNA expression and clinicopathological features of patients with gastric cancer

In recent years, as the circRNAs researches progressed, relationships between circRNAs and human diseases have gradually emerged. Through in-depth analyses and comparisons of circRNAs expression profiles in different diseases, it has become an indisputable fact that circRNAs can present different expression profiles in different diseases. Currently, many researches have listed out the expression profiles of circRNAs in GC^60^. Comparing the GC tissues with adjacent tissues through microarray technique, Sui,W et al. discovered 1285 differentially expressed circRNAs 61. Moreover, Dang,Y. et al's research reported the circRNAs expression profiles using five GC tissues and five matched non-cancer tissues with circRNA chip technique, and revealed that totally 713 circRNAs (191 up-regulated and 522 down-regulated) were differentially expressed^62^. In spite of the results of circRNAs expression profiles obtained by different experimental techniques were distinct, they indicated that masses of circRNAs expression levels changed during the development of GC^63^-^65^.

A study based on the results of microarray screening and qRT-PCR assay reported that the expression of hsa_circ_0000190 in GC tissues was down-regulated compared with that in adjacent normal tissues^66^. To assess the diagnostic value of hsa_circ_0000190, they generated a receiver operating characteristic (ROC) curve, observing that areas under the curve (AUC) were 0.60 and 0.75 in plasma and tissue, respectively. Moreover, the AUC, sensitivity and specificity of the combined group (combining plasma hsa_circ_0000190 and tissue hsa_circ_0000190) were 0.775, 0.712 and 0.750, respectively^66^. Further analyzing the association of hsa_circ_0000190 expression levels in GC tissue and plasma with the clinicopathological features of 104 GC patients, we discovered that the hsa_circ_0000190 expression levels were not only related to tumor size, lymph node metastasis and CA19-9 level, but also significantly correlated with CEA level, distal metastasis and TNM stage^66^. Results indicated that higher hsa_circ_0000190 levels in tissue were observed in GC patients with large tumor diameter (d≥5), no lymph node metastasis, no distant metastasis, low TNM stage and CA19-9 negative, while CEA positive patients had a higher hsa_circ_0000190 levels in plasma^66^.

Another research revealed that hsa_circ_0001895 was also correlated with clinicopathological features of GC patients^67^. By analyzing the relationships between the clinicopathological features of 96 GC patients and hsa_circ_0001895 expression level in GC tissues, Shao,Y et al, found that the expression level of hsa_circ_0001895 was high in patients with well differentiation, Borrmann grade III and IV, and GEA positive expression^67^.

Furthermore, Lu,R et al concerned with hsa_circ_0006633, detecting its expression level in GC cell line, tissue and plasma through qRT-PCR method and discovered it could act as a tumor suppressor molecule^68^. In healthy tissues, the expression of hsa_circ_0006633 was significantly higher than that in gastritis and atypical hyperplasia tissues, and it was significantly higher in paracancerous tissues than in GC tissues. Concerning the relationships between the expression level of hsa_circ_0006633 and clinicopathological features in 96 patients with GC, they elucidated that patients without distant metastasis and CEA positive patients had statistically higher expression level of hsa_circ_0006633^68^.

It will contribute to identifying novel molecules involved in the GC development by analyzing the correlations of differentially expressed circRNAs with the clinicopathological features of patients with GC and helps us in better recognizing the progression of GC.

## CircRNAs can be used as a marker for early diagnosis of gastric cancer

CircRNAs can potentially be biomarkers for clinical disease diagnosis due to their more stable structure and longer half-life than linear RNAs, better abundance and high specificity. Of note, circRNAs in extracellular fluid (such as saliva and plasma) released through EVs and cell lysis are also abundant and specific, making circRNAs potentially useful as non-invasive biomarkers for clinical disease diagnosis. For GC patients, circRNAs in their tissues and plasma are expected to be biomarkers for early diagnosis of GC 12, 69, 70.

For hsa_circ_002059, researchers suggested that it was significantly down-regulated in GC tissues and was correlated with distant metastasis and TNM stages through detecting 96 pairs of GC tissues and their corresponding normal gastric tissues by using qRT-PCR method^71^. Moreover, as a diagnostic marker for GC, the AUC of hsa_circ_002059 was 0.73, and the sensitivity, specificity and cut-off value were 0.81, 0.62 and 12.9, respectively^71^. Another research performed by Zhao Q. et al used qRT-PCR method to observe the expression level of hsa_circ_0000181 and indicated that it was significantly down-regulated in GC tissues and plasma of GC patients than in paired normal tissues and healthy people^72^. Additionally, analyses on its diagnostic value showed that the hsa_circ_0000181 level in GC tissue and plasma could act respectively as invasive and non-invasive biomarker for diagnosing GC^72^. Observing the diagnostic ability of hsa_circ_0000181 in GC tissues and in plasma samples, we found that the specificity of tissue specimens was 0.852 which was significantly higher than that of plasma samples 0.206; the sensitivity of tissue specimens was 0.539 while sensitivity of plasma samples was 0.990^72^. Therefore, plasma circRNAs, as diagnostic biomarkers, have advantages over tissue circRNAs in its non-invasiveness and better diagnostic ability for early GC.

In conclusion, the sensitivities of hsa_circ_002059 detected in both tissue samples and plasma samples meet the requirements of the diagnosis biomarker for GC^71^. CircRNAs have vast application prospect in non-invasive diagnosis and early diagnosis of GC. The diagnostic efficiency of circRNAs as a biomarker for gastric cancer is shown in Table 2.

## CircRNAs provide new specific targets for the treatment of gastric cancer

Recently, as digging deeper into the circRNAs, researchers have a heightened awareness of their functions. Up-regulated or down-regulated circRNAs may be novel specific therapeutic targets for GC with different phenotypes, invasion or metastasis ability, improving the survival rate of patients with GC.

Firstly, circRNAs can regulate the malignant phenotype related signaling pathway of GC through some specific functions. Hence, regulating the level of circRNAs may steer GC cells into a better condition. CiRS-7, as the firstly discovered circRNA with miRNA sponge function represses the miR-7 function in GC tissues^73^. Up-regulated ciRS-7 regulates the PTEN/PI3K/AKT pathway by suppressing miR-7, consequently affecting the apoptosis and migration of GC cells^73^. In addition, down-regulated hsa_circ_0000096 in GC tissues can impact the proliferation and migration of GC cells by modulating cyclin D1, CDK6, MMP-2 and MMP-9^74^. Inhibited hsa_circ_0000096 significantly suppress the proliferation and migration of GC cells^74^. Besides, inhibition of hsa_circ_0047905, hsa_circ_0138960 and hsa_circRNA7690-15 not only down-regulates the expressions of maternal genes, but also represses the proliferation and invasion of GC cells, discovered in another study on GC 75. Based on these reported findings, researchers suggest that the inhibited intracellular circRNAs result in suppressed proliferation and invasion abilities of GC cells, thus attaining a better phenotype of GC. This phenomenon can also be observed in animals. For instance, the growth rate of mice injected with miR-7 over-expressed tumor cells is lower than that of mice injected with ciRS-7 over-expressed tumor cells. The inhibition of hsa_circ_0000096 in nude mice can induce the decrease of Ki67 and VEGF.

Secondly, EVs make it possible for circRNAs to be applied to targeted therapy of GC. As a membranous vesicle, EVs can encapsulate specific biomolecules, participating in intracellular communications. Current studies have elucidated that circRNAs can be wrapped by EVs. Like circRNAs in cells, circRNAs exist in homeostasis in exocrine under normal conditions. In diseased conditions, however, homeostasis in exocrine is off, as the balance of intracellular circRNAs shifts. By modifying the number of EVs and specific circRNA levels in EVs, the content of circRNAs in receptor cells can be regulated which restores the homeostasis of the intracellular circRNAs, amending the downstream signaling pathways, such as miRNAs, so that the biological behaviors of receptor cells can be improved. These circRNAs play various roles in different periods of GC development through specific pathways. Compared with traditional therapies, these therapies using tissue and developmental stage specific circRNAs as targets for drugs or drug treatments act on particular targets, obtaining a more targeted way, which can not only help prevent side effects but also conduct individualized treatment for the distinct conditions of individual disease.

## Correlation between circRNAs expression levels and prognosis in patients with gastric cancer

Recent studies have demonstrated that circRNAs not only affect the proliferation abilities of GC cells through regulating signal pathways and are related to different phenotypes, but also have the ability to predict the relapse free survival and overall survival of patients^76^. At present, biomarkers circPVT1, circ_100269, etc have been discovered in GC tissues to be associated with GC prognosis.

CircPVT1 encoded by PVT1 gene was up-regulated in GC tissues, and was illustrated to be a proliferative factor for GC in further cell experiments^77^. Analyzing the expression of circPVT1 in 187 GC patients and their 85 months follow-up results, researchers revealed that circPVT1 was an independent prognostic biomarker for the overall survival (OS) rate and disease-free survival (DFS) of patients with GC^77^. Another study indicated that circ_100269 was associated with GC cell proliferation^78^. *In vitro* cell experiment, expression of circ_100269 was found to be inversely correlated with miR-630 and a direct interaction exist between them according to the results of dual-luciferase assay^78^. What's more, overexpressed circRNA_100269 could repress cell proliferation^78^. In tissue experiment, circRNA_100269 was downregulated in GC tissues, meanwhile demonstrating a prominent predictive function for the overall survival of the patients 78. All these findings suggested that circPVT1 and circRNA_100269 might not only play roles in the treatments of GC, but also be prognostic biomarkers for GC. Correlation between circRNAs and the prognosis of gastric cancer is shown in Table 3.

## Conclusion

CircRNA, as a kind of tissue specific RNA with more stable structure than linear RNAs play vital roles in physiological processes, pathological processes and disease progression. This review summarized CircRNAs and its relationship with gastric cancer from multi-angle of view. Evidence-based results suggests that circRNAs are not only involved in the regulation of the biological behaviors of GC cells, such as proliferation, invasion, migration and EMT, but also related to the clinicopathological features of GC patients, such as tumor differentiation, TNM stage and metastasis. CircRNAs are also regarded as biomarkers for early diagnosis and prognosis of GC. CircRNAs participating in the development of GC have the potential as novel targets for the individualized treatment of GC. The molecular mechanism explaining the correlations between circRNAs and GC is a emerging field and further researches are in demand. Furthermore, as biomarkers for the diagnosis and prognosis of GC, it's of vital significance to further screen and verify circRNAs in tissues and plasma.

## Figures and Tables

**Table 1:** CircRNAs associated with gastric cancer

Role	CircRNAs	circRNA ID	Gene Symbol	Chromosome	Best transcript	Function	Mechanism	Year	Ref.
**Oncogene**	circPVT1	/	PVT1	chr8	/	proliferation	as a sponge for members of the miR-125 family	2017	77
	hsa_circ_0047905	hsa_circ_0047905	SERPINB5	chr18	NM_002639	proliferation	positively correlated with their parental gene mRNA	2017	75
	circNRIP1		NRIP1	chr21	NM_003489	proliferation	as a sponge for members of the miR-149-5p to affect the	2019	47
						migration	expression level of AKT1		
						invasion			
	circDLST	has_circ_0032627	DLST	chr14	NM_001933	proliferation	as a sponge for members of the miR-502-5p to activate	2019	46
						invasion	the NRAS/MEK1/ERK1/2 signaling		
	hsa_circ_0067997	hsa_circ_0067997	FNDC3B	chr3	NM_022763	invasive	regulating miR-515-5p/XIAP axis	2019	48
	circ-ERBB2	/	/	/	/	proliferation	sponged miR-503 and miR-637	2019	54
						apoptosis			
						migration			
						invasion			
	circRNA_001569	/	/	/	/	apoptosis	suppressing the expression of miR-145, which was mediated by NR4A2	2019	50
	circPDSS1	/	/	/	/	apoptosis	regulating miR-186-5p and NEK2,	2019	49
	circ_DONSON	/	DONSON	chr21	/	proliferation	recruiting the NURF complex to initiate SOX4 expression	2019	55
						migration			
						invasion			
						apoptosis			
**Suppressor**	circRNA_100269	/	LPHN2	chr1	/	proliferation	the target miRNA was miR-630	2017	78
	circ-104916	/	/	/	/	proliferation	downregulated E-cadherin,upregulated,N-cadherin, Vimentin and Slug	2017	56
						migration			
						invasion			
						metastasis			
	hsa_circ_0000096	hsa_circ_0000096	HIAT1	chr1	NM_033055	proliferation	regulating cyclin D1, CDK6, MMP-2 and MMP-9	2017	74
						migration			
	circLARP4	/	LARP4	chr12	/	invasion	sponging miR-424 and upregulating LATS1 gene	2017	57
	circ-ZFR	/	/	/	/	proliferation	sponging miR-130a/miR-107 and regulating PTEN	2018	25
						apoptosis			
	hsa_circ_0001368	hsa_circ_0001368	KLHL24	chr3	NM_017644	proliferation	miR-6506-5p/FOXO3 axis	2019	58
						invasion			
	circFAT1 (e2)	hsa_circ_0001461	FAT1	chr4	NM_005245	proliferation	regulating the miR-548g/RUNX1 axis in the cytoplasm and targeting YBX1 in the nucleus	2019	59
						migration			
						invasion			

**Table 2:** Diagnostic efficiency of circRNAs for gastric cancer

CircRNAs	Sample	Expression	AUC	Se	Sp	cut-off	Year	Ref.
hsa_circ_0047905	tissue	up-regulation	0.850	0.743	0.877	/	2017	75
hsa_circ_0138960	tissue	up-regulation	0.647	0.677	0.645	/		
hsa_circRNA_7690-15	tissue	up-regulation	0.681	0.679	0.613	/		
hsa_circ_102958	tissue	up-regulation	0.740	/	/	/	2019	60
hsa_circ_002059	tissue	Down-regulation	0.730	0.810	0.620	12.900	2015	71
hsa_circ_0000096	tissue	Down-regulation	0.820	0.880	0.560	12.900	2017	74
hsa_circ_0001649	tissue	Down-regulation	0.834	0.711	0.816	0.227	2017	11
hsa_circ_0001895	tissue	Down-regulation	0.792	0.678	0.857	9.530	2017	67
hsa_circ_0014717	tissue	Down-regulation	0.696	0.594	0.813	12.140	2017	10
hsa_circ_0003159	tissue	Down-regulation	0.750	0.852	0.565	12.310	2017	69
hsa_circ_0006633	tissue	Down-regulation	0.741	0.600	0.810	8.170	2017	68
hsa_circ_0000190	tissue	Down-regulation	0.750	0.721	0.683	6.830	2017	66
hsa_circ_0000190	plasma	Down-regulation	0.600	0.414	0.875	3.070		
hsa_circ_0000181	tissue	Down-regulation	0.756	0.539	0.852	9.400	2017	72
hsa_circ_0000181	plasma	Down-regulation	0.582	0.990	0.206	7.270		
hsa_circ_0000745	plasma	Down-regulation	0.683	0.855	0.450	/	2017	12
hsa_circ_0001017, hsa_circ_0061276	plasma	Down-regulation	0.966	0.955	0.957	/	2018	70
hsa_circ_0000467	plasma	up-regulation	0.790	0.705	0.648	/	2019	76

**Table 3:** CircRNAs and gastric cancer prognosis

CircRNAs	Sample	Expression	Number of cases	Survival	Follow-up (months)	HR (95%CI)	Year	Ref.
circPVT1	tissue	Up-regulation	187	OS	83	0.600(0.400-0.880)	2017	77
ciRS-7	tissue	Up-regulation	102	OS	60	2.110(0.940-3.890)	2018	73
ciRS-7	tissue	Up-regulation	154	OS	60	2.630(1.230-5.550)		
circRNA_100269	tissue	Down-regulation	112	OS	50	0.600(0.350-1.020)	2017	78
circLARP4	tissue	Down-regulation	80	OS	108	0.502(0.240-1.048)	2017	57
circPVT1	tissue	Up-regulation	187	DFS	85	0.490(0.330-0.720)	2017	77
